# Remarks on Tetanus

**Published:** 1831

**Authors:** 


XXVIII.

Remarks on Tetanus. By Dr. Sym.

These remarks are called forth in consequence of a paper on the same subject in the 13th number of the Glasgow Medical Journal, by Dr. Perry, analyzed in our 29th number, page 174. The following are the criticisms of Dr. Sym.

"Morbid anatomy is not the only source from which we may derive a knowledge of the seat of diseases; physiology often enables us to point out what organs are in fault, when certain functions are disturbed, because we know upon what organs the healthy performance of those functions depends. Now, the symptoms of traumatic tetanus confine themselves so exclusively to the muscles, that it appears to me difficult to avoid the theoretical conclusion, that local injuries of the motory organs produce the disease, by throwing into a state of morbid irritation the common origin of the nerves of the motory system. I say morbid *irritation*, because we are not warranted either by the symptoms of the disease, or dissection, to affirm that inflammation is present in every case, or in every stage of tetanus: the vascular excitement, of which traces are found in the vertebral canal, is more likely to have been the effect than the cause of that irritation which is communicated to the motory system of nerves, in the same manner as over-exertion of the mind causes increased action of the blood-vessels of the brain. I would not, however, rest the opinion I entertain, that the profession actually possesses sufficient grounds upon which to found a rational treatment of tetanus, merely upon principles deduced from the doctrines of physiology. Dr. Perry appears to me to have undervalued the facts observed by others, as well as by himself, respecting the morbid anatomy of the disease; for in his own two cases, in the valuable collection of cases published by Mr. Adams, in the 10th Number of this Journal, and in the two fatal cases contained in my collection in the preceding Number, the *post mortem* observations confirm the conclusion, that morbid irritation of the spinal cord had existed during life. Dr. Perry has added an interesting appendix to this proposition, by shewing a connexion between the morbid changes within the vertebral column, and similar changes in the condition of the nerves proceeding from the wound: and although, in his first case, he states, that the cutaneous nerves were those in which traces of inflammation were detected, I must remark, that the superficial peroneal nerve, which he particularizes, is principally expended upon the extensor muscles; and, as it lies between the muscular fascia and integuments of the lower and outer part of the leg, it must have been implicated in the sloughing and inflammation at the seat of the injury.

What, then, are the means by which we may arrest this morbid action of the nervous system of the muscles, or restrain it within safe bounds until it has wasted its force? I believe that when one portion of a system of organs, engaged in performing the same or similar functions, is irritated, the remaining portions partake of the irritation by sympathy; and that when the irritation of the former is soothed, the latter partake in the relief. I believe also, that when an undue determination of nervous excitement has been directed to one system of organs, other systems, performing dissimilar functions, sustain a diminution of nervous vigour; and that the excitement of the former may be allayed by irritating the latter. I fear we have few direct means of allaying irritation in the whole or any part of the muscular system, which are not liable to the objection of producing a still greater degree of torpidity in the secretory organs. Perhaps the warm bath, as a general remedy, and mild applications to the wound, or, if a slough exists, a fermenting poultice, to counteract, by its carbonic acid, the tendency which dead matter has to fall into putrid decomposition, comprise the most useful sedatives: opiates ought not to be employed to such an extent as to suspend the action of the other remedies. But in our choice of counter-irritants we have a much wider scope, and in severe cases we ought to take advantage of the full range. The skin, the mucous membrane lining the alimentary canal and urinary passages, and the salivary and biliary systems, furnish instruments which may be employed advantageously for the purpose of withdrawing from the muscular system a portion of nervous irritation. There are, however, certain states of the constitution, in which this transfer of irritation is more easily effected than in others. It is effected with less certainty when the system is in full vigour, than when reduced. In inflammation of the serous membranes, for instance, in plethoric subjects, if we apply a powerful blister to the skin before the force of the circulation has been broken by copious blood-letting, we are more apt to aggravate the inflammation than to relieve it; but the same remedy has most happy results, when applied after adequate depletion has deprived the constitution of the means of supporting two extensive foci of irritation at the same time. Unless, therefore, a tetanic patient is already in a proper state for counter-irritation, blood-letting ought to precede the use of local stimulants, and it ought to be repeated at short intervals until a decided reduction of vascular action has been obtained. Blisters and tartar-emetic ointment applied along the course of the spine extensively and unmercifully, (if I may use the expression,) so as to ensure a severe and long-continued irritation, stimulating embrocations, antimonials, and the frequent use of the warm-bath; purgatives of such a nature as stimulate more the mucous than the muscular coat of the alimentary canal, in which view croton oil is less eligible than oil of turpentine or neutral salts; calomel, to excite the biliary and salivary secretions; irritation of the urinary passages by setons or bougies, or by cantharides applied to the denuded surface of the cutis, or by the internal use of oil of turpentine;—these constitute the means of counter-irritation, which have hitherto been employed with greatest success, and which, I trust, if employed consistently, energetically, and in combination with each other, will, ere long, deprive this frightful disease of more than half its terrors."*

---

### XXIX.

**CHRONIC TUMOUR IN THE ABDOMEN, PRODUCED BY A LARGE ACCUMULATION IN THE SUPERIOR PORTION OF THE COLON, &c. By JOHN HOWSHIP, Esq.**

JULY, 1830, I had the opportunity of examining the body of a tall, stout woman, aged 72, who after many months confinement to bed had died, with the usual symptoms of disease in the uterus.

Upon enquiry of those who had attended upon and nursed the patient, I

* Glasgow Med. Journal, No. XIV. p. 202.

